# Hypothalamic GPCR Signaling Pathways in Cardiometabolic Control

**DOI:** 10.3389/fphys.2021.691226

**Published:** 2021-06-28

**Authors:** Yue Deng, Guorui Deng, Justin L. Grobe, Huxing Cui

**Affiliations:** ^1^Department of Neuroscience and Pharmacology, University of Iowa Carver College of Medicine, Iowa City, IA, United States; ^2^Department of Physiology, Medical College of Wisconsin, Milwaukee, WI, United States; ^3^Department of Biomedical Engineering, Medical College of Wisconsin, Milwaukee, WI, United States; ^4^Comprehensive Rodent Metabolic Phenotyping Core, Medical College of Wisconsin, Milwaukee, WI, United States; ^5^Iowa Neuroscience Institute, University of Iowa Carver College of Medicine, Iowa City, IA, United States; ^6^FOE Diabetes Research Center, University of Iowa Carver College of Medicine, Iowa City, IA, United States; ^7^Obesity Research and Educational Initiative, University of Iowa Carver College of Medicine, Iowa City, IA, United States

**Keywords:** hypothalamus, signaling pathways, hypertension, obesity, G protein-coupled receptors

## Abstract

Obesity is commonly associated with sympathetic overdrive, which is one of the major risk factors for the development of cardiovascular diseases, such as hypertension and heart failure. Over the past few decades, there has been a growing understanding of molecular mechanisms underlying obesity development with central origin; however, the relative contribution of these molecular changes to the regulation of cardiovascular function remains vague. A variety of G-protein coupled receptors (GPCRs) and their downstream signaling pathways activated in distinct hypothalamic neurons by different metabolic hormones, neuropeptides and monoamine neurotransmitters are crucial not only for the regulation of appetite and metabolic homeostasis but also for the sympathetic control of cardiovascular function. In this review, we will highlight the main GPCRs and associated hypothalamic nuclei that are important for both metabolic homeostasis and cardiovascular function. The potential downstream molecular mediators of these GPCRs will also be discussed.

## Introduction

Obesity, a pathological state characterized by an excessive accumulation of body fat resulting from a chronic positive energy balance, continues to be a major global public health problem leading to an enormous economic burden. Obese adults are at an elevated risk of developing comorbidities including type II diabetes mellitus and hypertension (Flegal et al., 2010). Despite decades of effort dedicated to mitigating the increasing obesity prevalence worldwide, the World Health Organization (WHO) estimated that approximately 39% of the global population is now either overweight or obese (Gonzalez-Muniesa et al., 2017). In addition to diet and lifestyle modifications, the development of efficacious and safe anti-obesity pharmaceuticals remains an important weapon against the obesity pandemic. Evidence from human genome-wide association studies (GWAS) and experimental data from animal models have identified several potential candidates for anti-obesity therapeutics, including signaling molecules comprising a class of cell surface receptors known as G-Protein Coupled Receptors (GPCRs). GPCRs are important drug targets, as approximately 34% of all Food and Drug Administration (FDA) approved drugs are targeted against these receptors (Hauser et al., 2018).

G-Protein Coupled Receptors are seven-pass transmembrane domain cell surface receptors that initiate downstream signaling cascades upon binding to designated ligands, which ultimately translate into a specific adaptive cellular response. The type of response evoked by an individual ligand is dictated by the coupling of activated GPCR to distinct downstream G protein(s). G proteins are heterotrimeric guanine nucleotide-binding complexes which function as signal transducers to relay and amplify extracellular signals from ligand-activated GPCRs. These heterotrimeric complexes are composed of three subunits: G_α_, G_α_, and G_γ_. There are four major subtypes of G_α_ (G_αq/11_, G_αi_, G_αs_, and G_α12_), five different subtypes of G_α_ and twelve G_α_ subunits (Smrcka, 2008; Kostenis et al., 2020). GPCR activation results in the exchange of guanosine-diphosphate (GDP) to guanosine-triphosphate (GTP) on the G_α_ subunit, which promotes the dissociation of the G_α_ and G_α_ G_α_ complexes to initiate downstream signaling. The G_αq_ subunit signals through calcium and protein kinase C (PKC) signaling pathways by activating phosphoinositide phospholipase C beta (PLC-β), whereas G_αs_ stimulates and G_αi_ inhibits cyclic adenosine monophosphate (cAMP)-dependent signaling by modulating adenylyl cyclases activity (Neves et al., 2002). In addition to signaling by the G_α_ subunits, the G_α_ G_α_ subunits also appear to be capable of transducing downstream signals, further diversifying the intracellular responses initiated by a GPCR (Senarath et al., 2018; Smrcka and Fisher, 2019).

GPCR signal transduction can be regulated by Regulators of G-protein Signaling (RGS) proteins as well as β-arrestins. RGS proteins function as terminators of G protein signaling by enhancing the intrinsic GTPase activity of Gα subunits to facilitate the GTP to GDP hydrolysis, thereby promoting the reassociation between the G_α_ and G_α_ G_α_ subunits (Perschbacher et al., 2018). Thus, RGS proteins act to modulate the duration and subsequent termination of activated GPCR signaling. An additional layer of GPCR signaling regulation is provided by scaffold proteins such as β-arrestins, which include two subtypes, β-arrestins-1 and -2 and G protein-coupled receptor kinases (GRKs) (Lohse et al., 1990; Attramadal et al., 1992; Lymperopoulos, 2018; Chaudhary and Kim, 2021). Upon ligand induced receptor activation, GRKs are recruited to and phosphorylate activated GPCRs, subsequently allowing for β-arrestins to bind and promote the termination of further G-protein mediated signaling by inducing receptor internalization as well as potentially forming a signaling complex with the mitogen-activated protein kinases ERK1/2 to transduce ligand-specific downstream signaling (Tian et al., 2014; Eishingdrelo et al., 2015). While both β-arrestin-1 and β-arrestin-2 share a high degree of sequence and structural similarity and exhibit similar expression and localization patterns, increasing studies have shed light on functional distinction between these two β-arrestin subtypes [reviewed in Srivastava et al. (2015)]. Multiple lines of evidence from human genetic analysis as well as experimental data implicated critical roles for G proteins signaling in cardiometabolic regulation. For example, it has been reported that patients with single nucleotide polymorphisms (SNPs) in the gene encoding G_αs_ (GNAS) are associated with increased risk of sudden cardiac death, whereas loss-of-function mutations within GNAS are associated with early-onset obesity (Wieneke et al., 2016; Hanna et al., 2018). Furthermore, mice deficient for G_αs_ or G_α*q/*11_ specifically in the dorsomedial hypothalamus (DMH) develop obesity (Chen et al., 2019; Wilson et al., 2021). Thus, functionally intact G protein signaling is essential for cardiometabolic homeostasis.

In this review, we will highlight several clinically targeted GPCRs located within the hypothalamus with well- described roles in cardiometabolic control and explore the potential G protein signaling cascade(s) these GPCRs employ to exert these cardiometabolic effects.

## Hypothalamic MC4R Signaling Pathways in Cardiometabolic Regulation

The melanocortin 4 receptor (MC4R) is a highly conserved GPCR widely expressed in the central nervous system (CNS), including the cortex, hippocampus, amygdala, hypothalamus, brainstem, and spinal cord, and the highest expression of MC4R has been observed in the paraventricular nucleus of hypothalamus (PVN) among other brain nuclei (Kishi et al., 2003; Liu et al., 2003; Staubert et al., 2007). PVN^MC4R^ neurons receive dense innervation from two distinct and counteracting populations of neurons in the arcuate nucleus of hypothalamus (ARC): catabolic proopiomelanocortin (POMC)-expressing neurons and anabolic agouti-related peptide (AgRP)-expressing neurons (Garfield et al., 2009; Gautron et al., 2015). Functionally, ARC^POMC^ neurons release MC4R agonist alpha-melanocyte stimulating hormone (α-MSH), leading to reduced food intake and increased energy expenditure (Kim et al., 2000; Atasoy et al., 2012; Zhan et al., 2013; Dodd et al., 2015), whereas ARC^AgRP^ neurons release the MC4R inverse agonist AgRP, leading to the opposite physiological outcomes (Aponte et al., 2011; Atasoy et al., 2012). MC4R mutations represent the most prevalent form of monogenic obesity (Farooqi et al., 2003) and both animal models and human patients carrying loss-of-function MC4R mutations exhibit early-onset obesity caused by hyperphagia and reduced energy expenditure (Bolze and Klingenspor, 2009; Kuhnen et al., 2019; Rene et al., 2021).

Further functional dissection of MC4R signaling in different brain regions has been achieved through conditional Cre-loxP recombination techniques. For example, the necessity and sufficiency of PVN^MC4R^ neurons in the regulation of feeding and body weight have been demonstrated through AAV-Cre-mediated postnatal deletion of MC4Rs specifically in the PVN of MC4R^loxP/loxP^ and reactivatable MC4R^loxTB/loxTB^ mice. AAV-Cre-mediated deletion of PVN^MC4R^ leads to hyperphagia and obesity (Garza et al., 2008), while PVN-specific MC4R re-expression markedly suppresses hyperphagia and body weight gain in obese MC4R-null (MC4R^loxTB/loxTB^) mice (Shah et al., 2014). Furthermore, the Sim1-Cre-mediated selective restoration of MC4R expression in the PVN of obese MC4R-null mice normalizes food intake and reduces body weight by ∼60% (Balthasar et al., 2005; Rossi et al., 2011; Sohn et al., 2013). Addtionally, AAV-Cre injection into the DMH of MC4R^loxP/loxP^ mice also significantly increased body weight likely due to decreased energy expenditure (Chen et al., 2017). Functional circuit mapping revealed that PVN^MC4R^ neurons make functional synaptic connections to the lateral parabrachial nucleus (LPBN) and optogenetic stimulation of PVN^MC4R^LPBN terminals inhibit feeding, indicating an important role of PVN^MC4R^-LPBN pathway in appetite regulation (Shah et al., 2014; Garfield et al., 2015). On the other hand, MC4R regulates energy expenditure and glucose metabolism through other distinct pathways. Previous retrograde trans-synaptic neuronal tracing studies have shown that interscapular brown adipose tissue (iBAT) is innervated by sympathetic premotor neurons that express MC4R in PVN, brain stem and intermediolateral cell column (IML) (Voss-Andreae et al., 2007; Song et al., 2008; Kooijman et al., 2014). Selective restoration of MC4R expression in cholinergic neurons of obese MC4R^loxTB/loxTB^ mice not only partially ameliorate obesity but also improve glucose homeostasis (Rossi et al., 2011; Sohn et al., 2013). We have previously shown that AAV-Cre-mediated specific re-expression of lateral hypothalamic area (LHA) MC4R signaling improves glucose tolerance in obese MC4R^loxTB/loxTB^ without affecting body weight (Morgan et al., 2015). The improved glucose uptake in these animals was mainly driven by elevated iBAT glucose uptake, accompanied by significantly increased sympathetic traffic to iBAT (Morgan et al., 2015). These studies underscore an important role of hypothalamic MC4R signaling in the regulation of metabolic homeostasis.

Hypothalamic MC4R signaling is also involved in the control of sympathetic outflow affecting cardiovascular function. While obesity is commonly associated with sympathetic overactivity and elevated blood pressure (BP), severely obese MC4R knockout mice exhibit normal to reduced sympathetic nerve activity (SNA) when compared to their lean wild-type littermates (Tallam et al., 2005). Conversely, chronic MC4R activation causes sustained increases in BP that can be prevented by adrenergic receptor blockade (Kuo et al., 2004). The importance of MC4R in modulating SNA and BP is further supported by observations in humans that severely obese individuals due to genetic MC4R deficiency exhibit lower BP and reduced 24-h Norepinephrine (NE) excretion compared to obese individuals with normal MC4R function (Maier and Hoyer, 2010). Morgan et al. (2015) PVN is one of the integrative centers for sympathetic cardiovascular control through its descending projections to the rostral ventrolateral medulla (RVLM) and spinal cord (Stocker et al., 2013). Microinjection of a synthesized MC4R agonist, melanotan II (MTII, an α-MSH analog), directly into PVN increases SNA and BP in an SNA- and MC4R-dependent manner (Kuo et al., 2003, 2004). In addition, sympathetic response to insulin also requires PVN^MC4R^ signaling and activation of RVLM neurons (Ward et al., 2011), and MC4R agonists depolarize RVLM-projecting pre-sympathetic PVN neurons (Ye and Li, 2011) which have been implicated in obesity-associated hypertension (Stocker et al., 2007). Moreover, pharmacological inhibition of MC4R restores normal BP in spontaneously hypertensive rats (SHR) with elevated SNA (da Silva et al., 2008). Consistent with these observations, our recent comprehensive anterograde tract-tracing revealed that PVN^MC4R^ neurons densely innervate the ventrolateral medulla (VLM) and thoracic spinal cord (TSC) that are critical for sympathetic activation, and chemogenetic activation of PVN^MC4R^ neurons not only suppresses feeding but also elevate BP and iBAT thermogenesis (Singh et al., 2021). These observations suggest the existence of distinct MC4R neuronal circuits for the regulation of different aspects of cardiometabolic homeostasis. Further fine-mapping of functional circuits is needed for a better understanding of hypothalamic MC4R signaling pathways and their physiological impact.

**FIGURE 1:**
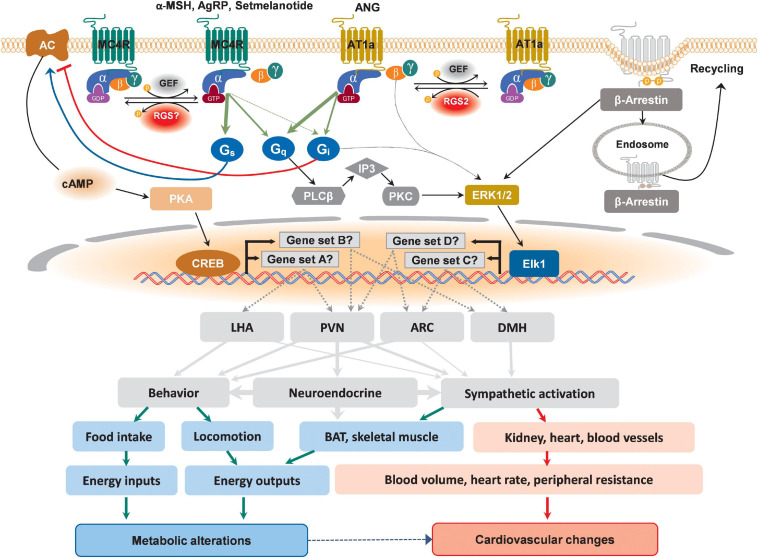
Schematic depicting possible divergent signaling cascades downstream of MC4R and AT1a in distinct hypothalamic nuclei that might differentially affect metabolic and cardiovascular regulations. Upon binding to its designated ligands, MC4R activates canonical G_αs_-AC-cAMP-PKA-CREB pathway, leading to the transcription of genes in different hypothalamic nuclei. Additionally, the activation of non-cannonical signaling pathways involving G_αq_ and G_αi_ has also been reported for MC4R, which lead to the activation of ERK-Elk1 transcriptional axis to drive the expression of different sets of responsive genes in different hypothalamic nuclei. Differential recruitment of these divergent signaling pathways may defines ultimate physiological changes. For AT1a, although reported canonical pathway is G_αq_-mediated activation of ERK, a possible non-canonical coupling to G_αi_ has also been supported experimentally, which, depending on the action of site, may lead to differential metabolic and cardiovascular outcomes. In addition to well-known receptor internalization for subsequent recycling or degradation, β-Arrestin-mediated activation of ERK transduction has also been reported for both MC4R and AT1a. These multilevel selectivities of GPCR signaling pathways ranging from intracellular molecular events to the brain regions of action and the effective organs could all lead to different metabolic alterations and cardiovascular changes.

The classical signaling pathway for the MC4R is by coupling to the heterotrimeric stimulatory G protein (G_αs_) which activates adenylyl cyclase to convert ATP to cAMP (Breit et al., 2011; Podyma et al., 2018). However, among all identified MC4R variants, only few have shown impaired cAMP level (Vollbach et al., 2017), which strongly suggests the existence of other signaling pathways. Indeed, more recent studies using the mouse hypothalamic cell line GT1-1 identified that MC4R can increase intracellular Ca^2+^ through the G_αq_/phospholipase C (PLC)-dependent signaling pathway (Newman et al., 2006). In another mouse hypothalamic cell line, GT1-7, α-MSH-induced G_α_ protein activation (by incorporation of GTPγS35) can be partially blocked by the G_αi_ inhibitor pertussis toxin (PTX), while the efficacy of α-MSH to induce cAMP production increased by 53%, indicating activation of both G_αs_ and G_αi_ signaling pathways (Buch et al., 2009). Interestingly, AgRP, which promotes feeding and suppresses energy expenditure when administered centrally (Small et al., 2001, 2003), was found to selectively activate G_αi_ signaling in these GT1-7 cells (Buch et al., 2009). It was recently reported that mice with homozygous G_αs_ deficiency in MC4R-expressing cells developed obesity due to both increased food intake and decreased energy expenditure, and the ability of the MC4R agonist MTII to stimulate energy expenditure as well as to inhibit food intake was significantly impaired in these animals (Podyma et al., 2018). Another recent publication demonstrated that the inhibitory effect of PVN-targeted microinjection of MTII on food intake is lost in mice lacking G_αq/11_, but not G_αs_, specifically in the PVN (Li et al., 2016). Interestingly, the BP response to MTII was absent only in animals lacking G_αs_ specifically in the PVN (Li et al., 2016). In line with these findings, setmelanotide, a recently FDA-approved MC4R agonist, exhibits clinically significant efficacy in reducing food intake and body weight without affecting BP, likely due to biased G_αq_ activation (Collet et al., 2017). Using a HEK293 cell-based system that incorporated a reporter gene, it is suggested that setmelanotide was about 80 times more potent than α-MSH for G_αq_/PLC activity (Clement et al., 2018). Furthermore, extracellular signal-regulated kinase 1/2 (ERK1/2) activation is also identified in cells expressing MC4R, which is thought to be involved in metabolic regulation. ERK1/2 activation through MC4R seems depending on different cellular contexts: NDP-α-MSH (an α-MSH analog) induces ERK1/2 activation via G_αi_ protein in HEK293 cells stably expressing MC4Rs, but through Ca^2+^/PKC pathway in GT1–1 cells with endogenous MC4R expression (Chai et al., 2006). Interestingly, several MC4R variants with normal cell surface expression, ligand binding and cAMP production are found to be ERK1/2 defective, which could account for the obese phenotype in these patients (He and Tao, 2014). These results are further supported by a recent publication that a gain-of-function human MC4R variant shows a signaling bias toward β-arrestin two recruitment and increased ERK1/2 activity (Lotta et al., 2019). These variants are protective against obesity and obesity-related cardiovascular diseases (Lotta et al., 2019). More studies are still needed to identify the *in vivo* MC4R-G_α_ coupling in different hypothalamic nuclei, the physiological outcome of different MC4R-G_α_ coupling, and the mechanisms behind biased MC4R signaling activation (Figure 1).

## The Brain Renin-Angiotensin System and the AT1A Receptor in Cardiometabolic Control

The renin-angiotensin system (RAS) is a hormone system that plays an essential role in the regulation of fluid and electrolyte balance, blood volume, systemic vascular constriction and BP, thereby establishing the RAS as a major controller of the cardiovascular system. The RAS exists as both a circulating hormone system as well as a localized, tissue-specific paracrine/autocrine/intracrine signaling system in organs such as the brain and adipose tissue. Angiotensin II (ANG), the canonical effector of the RAS, is the byproduct of angiotensinogen through a series of sequential enzymatic cleavages. ANG exerts its effect through the binding and activation of two known GPCRs – the angiotensin type I receptor (AT1, encoded by the *AGTR1* gene) and the angiotensin type II receptor (AT2, encoded by the *AGTR2* gene). Unlike humans, there are two AT1 isoforms in rodents - AT1a and AT1b, encoded by *Agtr1a* and *Agtr1b*, respectively (Elton et al., 1992; Iwai and Inagami, 1992; Sandberg et al., 1992). Early studies found that AT1a is critical for the BP response effects of the brain RAS, whereas the AT1b receptor is involved in the dipsogenic effects of central ANG actions (Davisson et al., 2000). Blockade of AT1a and AT1b receptors with the antagonist losartan in the paraventricular nucleus (PVN) prevents the reflex reduction in renal blood flow induced by elevated core body temperature, underscoring this receptor as a critical mediator of cardiovascular thermoregulatory responses (Chen et al., 2011).

In addition to the well-defined roles of the RAS in cardiovascular control, there is increasing evidence demonstrating a multimodal role for this hormone system in energy homeostasis, in part through affecting resting metabolic rate (RMR) (Littlejohn and Grobe, 2015). Interestingly, differential engagement of the AT1a and AT2 receptors by ANG elicits opposing actions on RMR, as brain AT1a activation increases RMR to stimulate energy expenditure, whereas adipocyte-specific activation of AT2 results in RMR suppression through inhibition of the beiging/browning process and thereby thermogenic capacity of these cells (Grobe et al., 2011; Littlejohn et al., 2016). The brain RAS is required for RMR control in response to multiple stimuli including high fat diet, leptin, and deoxycorticosterone acetate (DOCA)-salt (Grobe et al., 2011; Claflin et al., 2017). In addition to brain regions implicated in cardiovascular control such as the subfornical organ (SFO), supraoptic nucleus (SON), rostral ventrolateral medulla (RVLM) and organum vasculosum of the lamina terminalis (OLVT), AT1a expression is also observed in a subset of neurons expressing agouti-related peptide (Agrp) within the arcuate nucleus (ARC) of the hypothalamus, a region critical for the homeostatic regulation of energy balance by integrating the actions of peripheral metabolic hormones, such as leptin, insulin and ghrelin (Claflin et al., 2017; Sapouckey et al., 2017; Morselli et al., 2018; Sandgren et al., 2018; Deng and Grobe, 2019). Several studies have purported critical roles for intact AT1a signaling within the brain to mediate thermogenic SNA and RMR responses. For example, disruption of AT1a signaling by (1) central AT1a blockade by the AT1a receptor antagonist losartan, (2) whole-body *Agtr1a* knockout, and (3) inhibition of angiotensin-converting-enzyme (ACE, which is required to generate ANG) all resulted in abrogation of thermogenic SNA to acute leptin injections (Hilzendeger et al., 2012). Furthermore, we recently demonstrated that genetic deletion of AT1a specifically from either leptin receptor (LepR)- or agouti-related protein (AgRP)-expressing cells abolish the thermogenic SNA and RMR responses to leptin, high-fat diet (HFD), and deoxycorticosterone acetate (DOCA)-salt (Claflin et al., 2017). Given that pharmacological inhibition of the brain RAS diminishes BP responses to these stimuli, it was surprising that genetic disruption of AT1a signaling in LepR-expressing cells did not alter BP responses to DOCA-salt. In addition, AgRP neurons are undoubtedly implicated in the control of feeding behavior, yet AT1a deletion in LepR- or AgRP-expressing cells did not affect food intake. The PVN is an integrative center coordinating neural outputs for both metabolic and cardiovascular control and AT1a is enriched in PVN neurodendocrine neurons expressing corticotropin-releasing hormone (CRH) (Aguilera et al., 1995; Hurt et al., 2015). Others have demonstrated that the loss of AT1a signaling in the PVN does not alter baseline body weight and BP; however, these animals exhibit exaggerated weight gain on HFD challenge and blunted ANG- and stress-induced hypertensive responses, underscoring the importance of PVN^AT1a^ signaling in physiological responses to metabolic and behavioral challenges (Northcott et al., 2010; Fan et al., 2012; de Kloet et al., 2013; Wang et al., 2016). Collectively, these observations support a role for hypothalamic AT1a signaling in differential regulation of BP and body weight through mechanisms that differ based upon the specific cellular localization of AT1a (Haynes et al., 1999).

AT1a activation has been reported to be coupled to multiple G_α_ proteins, including G_αq_, G_α12/13_, and G_αi_, to transduce downstream second messenger signaling (Figure 1; Shirai et al., 1995; Shibata et al., 1996). This GPCR-G_α_ coupling appears to be both tissue- and cell-type-dependent, which determines the molecular, cellular, and physiological outputs of AT1a activation. For example, in vascular smooth muscle cells, AT1a is coupled to both G_αq/11_ and G_α12/13_ (Harris et al., 2007; Wirth et al., 2008). Furthermore, Sauliere and colleagues employed bioluminescence resonance energy transfer biosensors to demonstrate that ANG and the biased agonist [1Sar4Ile8Ile]-angiotensin II mediate the coupling of AT1a to G_αq/11_ and G_αi_ in HEK293T cells expressing exogenous AT1a as well as in primary cardiac fibroblasts with endogenous AT1a receptors (Sauliere et al., 2012). Rgs2, one of RGS family proteins, is a potent negative regulator of AT1a signaling in various cell types, and roles for Rgs2 and G_αi2_ in the modulation of energy homeostasis have been suggested from observations that mice lacking an RGS-sensitive G_αi2_ allele displayed increased energy expenditure, whereas Rgs2-null mice are resistant to weight gain (Huang et al., 2008; Matsuzaki et al., 2011; Nunn et al., 2011). Given that Rgs2 is reported to be a regulator of AT1a signaling which couples to G_αi_ and that the expression of all three of these molecules is observed in a subset of AgRP-neurons, it has been hypothesized that the AT1a-G_αi2_-Rgs2 signaling cascade within Agrp neurons is critical for the RMR stimulating effects but not the BP promoting effects of ANG [reviewed in Deng and Grobe (2019)]. Upon activation by its ligand, both β-arrestin 1 and β-arrestin 2 are recruited to AT1a receptors (Sanni et al., 2010). The central actions of β-arrestins in regulating energy homeostasis remain underexplored. However, a recent study by Pydi and colleagues indicated a beneficial metabolic role for central β-arrestin 1, as selective loss of β-arrestin 1 in AgRP neurons exhibited impaired glucose tolerance and insulin sensitivity when fed a high-fat diet (Pydi et al., 2020). Further, while GRKs, such as GRK2 and GRK5, have been reported to mediate ANG-induced AT1a receptor desensitization in tissue specific manner, the roles of central GRK play in maintaining energy balance through the AT1a signaling cascade are still poorly studied (Haack et al., 2012; Pollard et al., 2020).

Adverse sympathoexcitatory and hypertensive effects of MC4R agonists, such as LY2112688, have profoundly limited the therapeutic use and curtailed further development of first-generation MC4R agonists for obesity treatment (Greenfield et al., 2009; do Carmo et al., 2017). Thus, when considering central AT1a signaling as a potential target for anti-obesity therapeutics, it is important to underscore that ANG may activate divergent signaling pathways and/or engage various neural circuits to differentially control RMR and BP, as mice lacking AT1a in AgRP neurons failed to display an increase in RMR in response to HFD or DOCA-salt treatments while keeping BP response intact (Claflin et al., 2017). This biased agonism of the AT1a receptor confers potential anti-obesity treatment through stimulating RMR with minimal cardiovascular side effects. While AT1a expression has been observed in hypothalamic nuclei implicated in both cardiovascular and metabolic regulations, including the ARC, PVN, SON, and dorsal medial hypothalamus (DMH), the functional roles of these AT1a-expressing neurons and underlying neural circuits mediating cardiometabolic control remain largely underexplored and continue to be an active area of research.

## Serotonin and Hypothalamic Serotonergic Receptors in Cardiometabolic Regulation

The neurotransmitter serotonin (5-hydroxytryptamine; 5-HT) has broad physiological effects including modulating moods and promoting satiety (Yabut et al., 2019). These diverse effects are mediated by a myriad of 5-HT receptors and receptor subtypes expressed in mammals. There are seven known 5-HT receptor families, 5-HT_1–7_, all of them are GPCR, except for the 5-HT_3_ receptor, which is a ligand-gated Na^+^ and K^+^ cation channel (Nichols and Nichols, 2008). Due to the inability of 5-HT to pass through the blood-brain barrier, its synthesis must occur locally within the brain, using the amino acid tryptophan as a precursor molecule (Boadle-Biber, 1993). Early evidence supporting a role for 5-HT in food intake modulation comes from observations that (1) the anti-serotonergic drug cyproheptadine stimulates appetite in human as well as in mice and (2) treatment with the 5-HT releasing agent, fenfluramine, causes significant weight loss in obese patients (Bergen, 1964; Guy-Grand, 1995). However, follow-up studies on patients who had been treated with fenfluramine revealed adverse cardiovascular effects, with an increased risk for developing primary pulmonary hypertension (PPH) (Brenot et al., 1993; Souza et al., 2008). Given the expansive physiological effects 5-HT has and the diverse set of receptors it can engage, it is not unexpected that the fenfluramine confers some adverse side effects and underscores the need to critically examine the various functions mediated by each 5-HT receptor subtype. Indeed, it has been reported that the fenfluramine metabolite, (+)-norfenfluramine, induces vasoconstriction and pressor effects through the 5-HT_2A_ receptor, suggesting that PPH incidences observed in fenfluramine treated patients may be due to inadvertent activation of this 5-HT receptor subtype (Ni et al., 2007).

The satiety-promoting effects of 5-HT are mediated, in part, by the 5-HT_1B_ and 5-HT_2C_ receptors expressed in neurons of the ARC (Makarenko et al., 2002; Xu et al., 2008). Activation of 5-HT_2C_ by selective agonist BVT.X resulted in decreased acute food intake in both genetic and diet-induced models of obesity in mice, and this appetite-suppressing effect is upstream of the melanocortin pathway, as mice deficient in the melanocortin 3 (MC3)/MC4 receptors do not exhibit food intake reduction effects induced by 5-HT_2C_ agonism (Lam et al., 2008). On the other hand, mice with 5-HT_1B_ receptor deletion exhibit increased weight gain and pharmacological activation of this receptor by the receptor agonist CP-94,253 inhibits food intake (Lee and Simansky, 1997; Lucas et al., 1998). Additional studies revealed that the 5-HT_2C_ receptor is coupled to G_α*q/*11_ whereas the 5-HT_1B_ receptor is G_αi/o_ coupled [reviewed in Hoyer et al. (1994)]. Thus, engagement of different G protein coupling by these two serotonin receptors within the same hypothalamic nuclei appears to promote the satiety effects. However, additional studies are warranted to determine whether these two receptors signaling pathways occur within the same cell or different cellular populations of the ARC. The therapeutic potential of 5-HT_2C_ signaling pathway was highlighted by the clinical use of lorcaserin, a selective 5-HT_2C_ agonist approved by the Federal Drug Administration (FDA) in 2012 for the treatment of obesity. While initial results from Phase 2 trials hinted participants taking the drug may be at increased risk of cardiac fibrosis, subsequent analysis concluded that lorcaserin exhibited a similar cardiovascular safety profile to that of placebo (Bohula et al., 2018). However, subsequent assessment of patients taking lorcaserin revealed a higher incidence of cancer diagnosis, prompting the FDA to request the manufacturer to withdraw the drug from the United States market (Sharretts et al., 2020). In contrast to the 5-HT_2C_ and 5-HT_1B_ receptors, whereby the hypophagic effects of serotonin were mediated by agonism of these receptors, it is the antagonism of the 5-HT_6_ receptor that promotes weight loss. Indeed, mice carrying a non-functional mutant 5-HT_6_ receptor are partially protected from high-fat induced obesity due to decreased food intake (Frassetto et al., 2008). Furthermore, RNAi-mediated central knockdown of 5-HT_6_ receptor signaling similarly results in weight loss and reduced food intake as observed in 5-HT_6_ null mice (Woolley et al., 2001). Within the hypothalamus, the G_αs_-coupled 5-HT_6_ receptor is expressed in cells of the PVN, VMH, and ARC (Roberts et al., 2002; Fisas et al., 2006). Consistent with previous studies demonstrating a critical role for the 5-HT_6_ receptor antagonism in promoting satiety, pharmacological inhibition of 5-HT_6_ receptor-mediated signaling with the receptor antagonist SB-399885 reduced food intake in rats, in concurrent with increased neuronal activation in cells located in the PVN and the nucleus tractus solitarius (NTS) (Garfield et al., 2014).

A recent study employing single-cell RNA-sequencing identified eleven transcriptomically distinct serotonin neurons of the dorsal and median raphe nuclei, where a subset of these serotonin neuronal clusters co-expressing thyrotropin-releasing hormone innervate the hypothalamus (Ren et al., 2019). However, the neuroanatomical basis of serotonin-sensing neurons located within hypothalamic nuclei that mediate appetite control remains largely underexplored. Given the diverse physiological responses mediated by serotonin as relayed by a vast receptor superfamily, it is essential to delineate the contribution of each serotonin receptor subtype and the relevant neurocircuitry to more effectively develop anti-obesity therapeutics while minimizing unwanted side effects.

## Hypothalamic α-Adrenergic Receptors in Cardiometabolic Regulation

Norepinephrine is an important neurotransmitter that regulates a variety of CNS functions, including cardiovascular responses, neuroendocrine homeostasis, feeding and wakefulness (Shor-Posner et al., 1985; Camargo and Saad, 2001; Smythies, 2005; Mallick and Singh, 2011; Berridge et al., 2012). These actions of NE are mediated by adrenergic receptors (ARs), which can be divided into three types (α_1_, α_2_, and β) and nine subtypes (α_1A–C_, α_2A–C_, and β_1–3_) (Strosberg, 1993). Upon ligand binding, α_1_-ARs induce depolarization via G_αq_-mediated increase in intracellular Ca^2+^ levels and closure of G protein-coupled inwardly rectifying potassium channels (GIRKs), while α_2_-ARs induce hyperpolarization via G_αi_-mediated inhibition of voltage-gated Ca^2+^ channels (VGCCs) and opening of GIRKs (Hein, 2006). On the other hand, β-ARs activation is excitatory, which is mediated by G_αs_-coupled signaling cascade [reviewed in Marzo et al. (2009)]. The intracellular regulators of AR signaling has been identified recently. Among these components, GRK2 was found to be significant for SNA-mediated cardiovascular homeostasis. It was previously shown that adrenal catecholamine production is tightly regulated by GRK2-controlled α_2_-AR signaling, providing potential mechanism of SNS hyperactivation under pathological conditions like heart failure (Lymperopoulos et al., 2007; Jafferjee et al., 2016). Whether similar mechanisms exists in hypothalamic ARs, remains to be further investigated.

In the hypothalamus, α_1_ and α_2_, especially subtype α_1A_ and α_2A_ are the two most abundantly expressed ARs and both are involved in the regulation of cardiometabolic control (Scheinin et al., 1994; Day et al., 1997). Early histochemical analysis revealed that the medial hypothalamus is densely innervated by ascending NE projections from the brainstem (Leibowitz and Brown, 1980). However, the distribution of AR-expressing cells in the hypothalamus remain largely unclear, mostly due to the lack of available AR subtype-specific transgenic Cre lines. Recently, a novel adrenergic NTS-ARC pathway modulating food intake was identified (Aklan et al., 2020; Chen et al., 2020). It is reported that NTS tyrosine hydroxylase (TH)-expressing neurons densely innervate the ARC, and activation of NTS^TH^-ARC fibers promote feeding via NE release onto ARC^AgRP^ neurons that express α_1_ AR (Aklan et al., 2020). Despite the limited information of neural circuits consisting of AR-expressing neurons, the development of selective pharmacological agonists/inhibitors has greatly expanded our understanding of the physiological roles of hypothalamic α_1_ and α_2_ signaling pathways. Microinjection of α_2_ agonists into PVN elicits feeding, while α_1_ agonist injection suppresses food intake (Goldman et al., 1985; Wellman and Davies, 1991b). Consistent with the agonist studies, it is documented that PVN-specific inactivation of α_1_-AR signaling abolished the feeding suppression induced by systemic injection of α_1_ agonist (Wellman and Davies, 1991a). Moreover, systemic and PVN-specific injection of α_2_ antagonists suppressed food intake (Alexander et al., 1993). These findings not only demonstrate that PVN α_1_ and α_2_-ARs are involved in the regulation of feeding, but also suggest potential intracellular antagonistic mechanisms between these two receptors. In line with this working model, brain slice electrophysiological recordings of NE-responsive PVN neurons revealed that the excitatory effect of NE is mimicked by α_1_ agonist phenylephrine (PHE), while the inhibitory effect of NE is mimicked by α_2_ agonist clonidine (Wellman et al., 1993). Similar co-localization and antagonistic actions are observed in other brain regions, including NTS, DMV and VMH, suggesting that this mutual antagonistic mode of action of α_1_ and α_2_-AR is generalized across different brain regions (Young and Kuhar, 1980; Saad et al., 1984; Feldman and Felder, 1989). Antagonistic action between α_1_ and α_2_ receptors on feeding is versatile and tightly regulated. Early microdialysis studies revealed that extracellular NE within the PVN peaked at the beginning of the dark cycle, accompanied by a sharp rise in α_2_-AR expression (Bhakthavatsalam and Leibowitz, 1986; Jhanwar-Uniyal et al., 1986; Morien et al., 1995). In addition, this elevation of NE was significantly correlated with eating onset (Bhakthavatsalam and Leibowitz, 1986). Though it is known that α_1A_ and α_2A_ are the most abundant subtypes in the hypothalamus, the physiological roles of different α_1_ and α_2_ subtypes remain to be further investigated. A recent study using cell type-specific transcriptomics in ARC neurons revealed that NE activates NPY/AgRP neurons through α_1A_ receptors and inhibits POMC neurons via α_2A_ receptors (Paeger et al., 2017), which shed light on future subtype-specific functional and mechanistic studies. However, it is not clear whether ARs-mediated feeding control is independent of other critical metabolic regulators, such as MC4R.

Hypothalamic α_1_ and α_2_-ARs are also involved in the regulation of cardiovascular functions, mainly through the modulation of sympathetic tone that is regulated by different hypothalamic nuclei. Injection of the α_1_ agonist PHE into the PVN significantly increased HR and sympathetic outflow (Mendonca et al., 2018). Moreover, injection of an α_1_ agonist metaraminol into the median preoptic nucleus (MnPO) significantly increased BP (Llewellyn et al., 2012; Takahashi and Tanaka, 2017). On the other hand, microinjection of α_2_ agonist clonidine into the anterior hypothalamic nucleus (AHN) caused a rapid decrease in BP, indicating inhibited sympathetic outflows (Peng et al., 2003). Due to the lack of selective pharmacological tools to dissect the function of distinct subtypes of α_1_ and α_2_-ARs, transgenic animals that lack specific AR subtypes are generated. Mice with selective deficiency of α_1A_ receptors are hypotensive (Rokosh and Simpson, 2002). On the other hand, α_2A_ knockout mice are insensitive to α_2_ agonists-induced hypotensive effect (Altman et al., 1999), suggesting that α_2A_ subtype mediates the most pharmacological functions of α_2_ agonists and that centrally abundant α_2A_ subtype is critical for cardiovascular regulation. Future experiments should focus on conditional knockout of AR subtypes in different hypothalamic nuclei with either cell-type-specific Cre lines or viruses-mediated regional deletion to identify the relative contribution of different AR subtypes to cardiometabolic homeostasis and address how these regulatory pathways might be perturbed in the context of obesity.

Even though we are still far from fully understanding the roles of hypothalamic α_1_ and α_2_ subtypes in the modulation of cardiometabolic homeostasis, it is clear that their functions are context-dependent, and crosstalks exist between the AR signaling and other neurotransmitters. Future single-cell transcriptomic studies will help to reveal the molecular profile of hypothalamic AR-expressing cells, providing better insights into the next-generation pharmacological agents targeting AR subtypes for therapeutic use.

## GLP-1, Hypothalamic GLP-1 Receptor and Cardiometabolic Regulation

Glucagon-like peptide (GLP)-1 is a small peptide hormone derived from the preproglucagon protein by enzymatic cleavage processes. Although the preproglucagon gene is observed in the L cells of the intestine, α cells of the pancreas, and some neurons residing the caudal brainstem and hypothalamus, the major source of endogenous GLP-1 is the gut (Mojsov et al., 1986). GLP-1 enhances the glucose-stimulated insulin secretion as well as inhibits glucagon secretion (Nauck et al., 1993a,b). Subsequent studies further revealed an effect of GLP-1 on food intake and body weight control (Tang-Christensen et al., 1996; Turton et al., 1996; Naslund et al., 1999). The physiological effects of GLP-1 are mediated by the GLP-1 receptor (GLP-1R) localized to both the periphery and the brain (Dunphy et al., 1998). Overexpression studies using recombinant cell lines suggest that GLP-1R can signal through multiple G proteins, including G_αs_, G_α*q/*11_, G_αi1_, and G_αi2_ (Wheeler et al., 1993; Montrose-Rafizadeh et al., 1999). Recruitment and subsequent signaling through the scaffolding protein, β-arrestin 1, has also been reported, suggesting that activated GLP-1R is also capable of transducing G protein independent signals (Sonoda et al., 2008). However, the G protein dependent and independent mechanisms of GLP-1R-mediated signaling transduction may not be mutually exclusive, as β-arrestin 1 knockdown impaired GLP-1 stimulated insulin secretion, cAMP production, ERK1/2 phosphorylation, as well as suppressed the anti-apoptotic effects of GLP-1 in pancreatic β cell lines obtained from β-arrestin 1 knockout mice (Sonoda et al., 2008; Quoyer et al., 2010). Interactions between β-arrestin 2, GRK2 and activated GLP-1R have been proposed by Jorgensen et al. (2011). In this study, Jorgensen and colleagues employed bioluminescence resonance energy transfer assays to assess interactions between these three molecules and observed that β-arrestin 2 competes with GRK2 for interaction with GLP-1R. However, given these assasys were performed *in vitro*, the physiological relevance of these interactions must be carefully evaluated *in vivo* in regards to the potential impact on cardiometabolic effects.

Due to its beneficial insulinotropic effect, several pharmacological GLP-1R agonists have been successfully developed and marketed for the treatment of type 2 diabetes mellitus (T2DM). These GLP-1R agonists include liraglutide and semaglutide, which are structurally similar acylated forms of GLP-1 (Knudsen and Lau, 2019). Liraglutide has been formulated as a once weekly injection treatment for T2DM, which was first approved in Europe in 2009 and then in the United States in 2010 (Peters, 2013). Similar to liraglutide, semaglutide (approved for T2DM management in 2017 in the United States and 2019 in Europe) was initially formulated as a once weekly injection GLP-1R agonist. In 2020, however, a once daily oral formulation of semaglutide was approved for T2DM patients, allowing for an easy route of administration (Granhall et al., 2019). Experimental as well as clinical observations have also shed light on additional health benefits of GLP-1R agonists, including the promotion of weight loss by reducing food intake and a decrease in major cardiovascular adverse events (MACE) (Heuvelman et al., 2020; Qiu et al., 2020). The anorexigenic effects of GLP-1R activation have been observed in both rodents and humans and this appetite-suppressing effect is likely centrally mediated (Beiroa et al., 2014; Henderson et al., 2016). Flint and colleagues observed that when GLP-1 was infused simultaneously as the start of an energy-fixed meal in a small cohort of healthy human volunteers, a significant reduction in energy intake was observed and satiety was promoted compared to control subjects who received placebo (Flint et al., 1998). An increase in energy expenditure was also observed in T2DM patients treated with liraglutide for over a year (Beiroa et al., 2014). Thus, GLP1-R agonism appears to act on both arms of the energy balance equation to promote body weight loss. Retrospective analysis of clinical trials involving liraglutide also supports the clinical benefit of this GLP-1 agonist on promoting weight loss (Mehta et al., 2017). Due to the overwhelming evidence of the beneficial effects of liraglutide in weight management, it was approved for obesity treatment in 2014. A large-scale, multi-nation clinical trial was initiated in 2018 to assess the efficacy and safety of semaglutide for weight management in obese and overweight adults. The results of this trial were recently reported and found that semaglutide demonstrated superior body weight reduction in trial participants, as compared to placebo (Davies et al., 2021). The results of this clinical trial paved the road for another GLP-1R agonist to be on the short list of potential anti-obesity therapeutics for approval for use in the general public.

The precise molecular and cellular mechanisms as well as the neurocircuitry underpinning the anorexigenic effects of GLP-1 remain to be fully elucidated, but key insights can be gleaned from several mechanistic studies. As alluded to above, the GLP-1-induced anorexigenic effect appears to be centrally mediated, as central GLP-1 administration into the ARC, the LHA or the PVN all resulted in reduced food intake and body weight in rats (Beiroa et al., 2014). c-Fos induction was observed in these hypothalamic nuclei upon GLP-1 infusion, indicating neuronal activation. In line with these observations, Secher and colleagues found that peripherally administered liraglutide can directly activate POMC neurons and indirectly inhibit AgRP neurons of the ARC (Secher et al., 2014). Central infusion of the GLP1-R antagonist Exendin (9–39) into the ARC, but not the PVN, blocked the weight loss effect induced by liraglutide, leading the authors to suggest that the ARC is a critical hypothalamic nucleus mediating GLP-1 regulation of body weight (Secher et al., 2014). In another study, Sandoval and colleagues observed that when GLP-1 was centrally infused into the ARC or the PVN, a reduction in food intake was only observed in rats with GLP-1 infused into the PVN but not the ARC (Sandoval et al., 2008). Consistent with a role for PVN neurons in GLP-1 induced food intake suppression, Liu and colleagues reported that GLP-1 expressing NTS neurons projecting to corticotropin-releasing hormone (CRH) neurons of the PVN are required for the food intake suppression by GLP-1 (Liu et al., 2017). Taken together, these observations critically support the concept that brain region-specific GLP-1R signaling cascades differentially activated by GLP-1 and liraglutide are involved in the pleiotropic effects of this ligand-receptor system.

GLP-1 and GLP-1R agonists also exert cardioprotective effects. In both rodents and humans, GLP-1R is expressed in cardiomyocytes, vascular smooth muscle cells, and endocardium (Campos et al., 1994; Wei and Mojsov, 1995). GLP-1 treatment significantly reduced infarction size in an ischemia-reperfusion model through adenylyl cyclase, phosphatidylinositol 3-kinase (PI3K), and ERK1/2 signaling cascades, as applications of inhibitors for these signaling pathways abrogate the GLP-1-mediated cardio-protection (Bose et al., 2005). More importantly, a meta-analysis of clinical trials evaluating GLP1-R agonists, such as liraglutide and semaglutide, revealed a trend toward reduction in incidences of MACE, lending credence to the potential of new cardiovascular disease treatments (Kristensen et al., 2019). The reduction in MACE by the treatment of GLP-1 and GLP-1R agonists may be attributed to the antihypertensive effect of this pleiotropic peptide hormone. Indeed, the long-term treatment of GLP-1R agonists in hypertensive T2DM patients resulted in reduced BP (Ferdinand et al., 2014; von Scholten et al., 2015). The antihypertensive effect exerted by GLP-1 and GLP-1R agonists seems dependent, in part, on dopamine beta-hydroxylase^+^ (DBH^+^) neurons located in the NTS of brainstem, as liraglutide injection into the fourth ventricle attenuated the development of hypertension in SHR rats with concomitant induction of c-FOS expression in NTS DBH^+^ neurons, whereas the ablation of these NTS DBH^+^ neurons dampened the antihypertensive effects of liraglutide injection (Katsurada et al., 2019). The precise mechanism(s) of how GLP-1 exerts its cardio-protective effects, however, remain yet to be fully clarified, as GLP-1Rs are also expressed in immune and renal cells and may additionally influence these two tissue organs to exert its cardioprotective effect (Hadjiyanni et al., 2010; Crajoinas et al., 2011). However, since these topics are beyond the scope of this review, we would like to point interested readers to recently published reviews examining the role of GLP-1/GLP-1R agonists on the immune system and the kidney (Zietek and Rath, 2016; Vitale et al., 2020).

## Perspective

**FIGURE 2:**
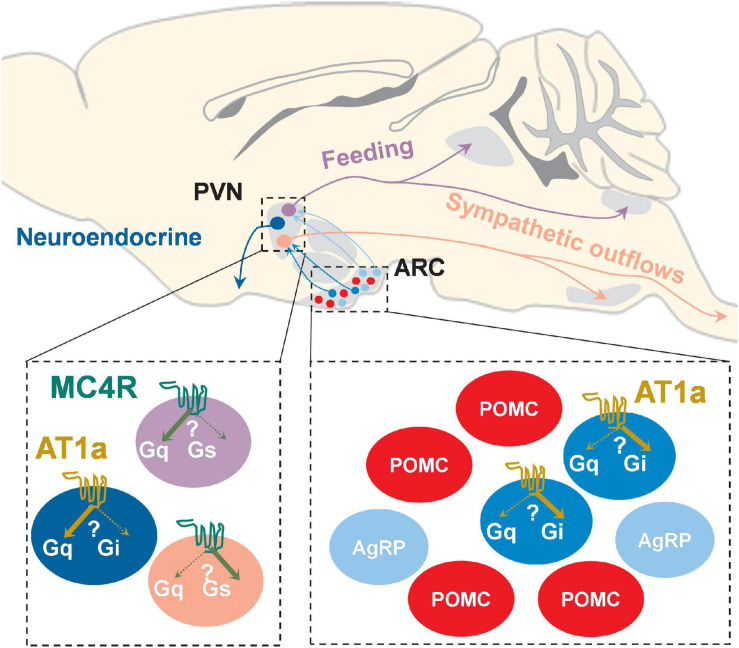
Schematic of possible brain circuit- and cell type-specific MC4R and AT1a signaling pathways in both PVN and ARC. PVN^MC4R+^ neurons project to different brain regions to differentially regulate feeding and sympathetic outflows, and it is possible that MC4Rs in these functionally and neuroanatomically distinct neurons may preferentially activate one involved downstream signaling cascade (G_αs_ and/or G_αq_) over another. AT1a is uniquely expressed in a subset of ARC AgRP^+^ neurons as well as PVN neuroendocrine neurons, and it has been suggested that AT1a in these neurochemically defined neurons may preferentially coupled to either G_αq_ (PVN) or G_αi_ (ARC AgRP^+^).

Despite enormous efforts in understanding the underlying pathogenesis of obesity, the development of safe and selective pharmacotherapeutics for obesity with minimal cardiovascular side effects has been unsatisfactory, largely due to the existence of divergent signaling pathways involved in both metabolic and cardiovascular regulations. The hypothalamus is crucial for orchestrating cardiometabolic homeostasis, and many GPCRs that are important for cardiometabolic regulation are expressed across different hypothalamic nuclei. As summarized in Figure 1 and Table 1, the coupling between the GPCRs and the G proteins, together with the neuroanatomical and cellular contexts, defines the physiological outcomes of activation and/or inhibition of a GPCR. Another layer of complexity in understanding the physiological roles of hypothalamic GPCRs comes from the possibly biased activation of signaling cascade in distinct types of hypothalamic neurons with either functionally segregated projection or unique molecular identity as modeled in PVN and ARC neurons using MC4R and AT1a as examples (Figure 2). Since the studies have shown that indiscriminate activation of sympathetic traffics to multiple metabolic and cardiovascular organs by various hypothalamic GPCR signaling might represent one of the common mechanisms for obesity-associated hypertension, unraveling the physiological roles of cell-type- and/or pathway-selective hypothalamic GPCR signaling is a critical step toward the future development of highly targeted therapeutics with minimal unwanted side effects, including brain circuit-specific GPCR activation and/or identification of biased agonism/antagonism for various GPCRs. Loss- and gain-of-function genetic approaches for a GPCR combined with functional circuit mapping may be necessary for a decisive evaluation of the physiological roles of a GPCR within a specific hypothalamic neurocircuitry.

**TABLE 1:** The downstream signaling effects of hypothalamic GPCRs involved in the cardiometabolic regulation.

GPCR	G protein	Effects upon G protein manipulation	References
MC4R	G_αq/11_	Signals through G_αq_ to increase intracellular Ca^2+^	*In vitro* (Newman et al., 2006)
		Setmelanotide preferentially activates G_αq_	*In vitro* (Clement et al., 2018)
	G_αs_	Signals through G_αs_ to increase α-MSH mediated cAMP production	*In vitro* (Buch et al., 2009)
		G_αs_ deficiency in MC4R-expressing cells promoted obesity development, increased food intake and decreased energy expenditure	*In vivo* (Podyma et al., 2018)
	G_αi_	Inhibition by pertussis toxin increased α-MSH mediated cAMP production	*In vitro* (Buch et al., 2009)
AT1a	G_αq/11_	Inhibition of G_αq_ signaling attenuated blood pressure increases due to renal artery stenosis and salt-induced hypertension	*In vivo* (Huang et al., 2008)
	G_αi_	ANG and [1Sar4Ile8Ile]-angiotensin II induced coupling resulted in decrease cAMP generation	*In vitro* (Sauliere et al., 2012)
5-HT2C	G_αq/11_	Signals through G_αq/11_ to stimulate phospholipase C activity and accumulation of inositol phosphates	*In vitro* (Hoyer et al., 1994)
5-HT1B	G_αi_	Signaling through G_αi_ inhibits adenyly cyclase activity	*In vitro* (Hoyer et al., 1994)
5-HT6	G_αs_	Signals through G_αs_ to promote cAMP production	*In vitro*, *In vivo* (Roberts et al., 2002; Fisas et al., 2006)
α1-AR	G_αq/11_	Signals through G_αq_ to increase intracellular Ca^2+^; closure of G-protein-coupled inwardly rectifying potassium channels (GIRKs)	Strosberg (1993)
α2-AR	G_αi_	Signals through G_αi_ to inhibit voltage-gated Ca^2+^ channels and opening GIRKs	Strosberg (1993)
GLP1R	G_αq/11_	Signals through G_αq_ to increase GLP-1 (7–37) mediated intracellular Ca^2+^	*In vitro* (Wheeler et al., 1993; Montrose-Rafizadeh et al., 1999)
	G_αs_	Signals through G_αs_ to increase GLP-1 (7–37) mediated cAMP production	*In vitro* (Wheeler et al., 1993; Montrose-Rafizadeh et al., 1999)
	G_αi_	Ligand activation increased GTP-azidoanilide incorporation into G_αi_	*In vitro* (Wheeler et al., 1993; Montrose-Rafizadeh et al., 1999)

## Author Contributions

YD and GD wrote manuscript. JG provided critical inputs and revised the manuscript. HC revised and finalized the manuscript. All authors contributed to the article and approved the submitted version.

## Conflict of Interest

The authors declare that the research was conducted in the absence of any commercial or financial relationships that could be construed as a potential conflict of interest.
